# The Readiness of ChatGPT to Write Scientific Case Reports Independently: A Comparative Evaluation Between Human and Artificial Intelligence

**DOI:** 10.7759/cureus.39386

**Published:** 2023-05-23

**Authors:** Maryam Buholayka, Rama Zouabi, Aditya Tadinada

**Affiliations:** 1 Oral and Maxillofacial Radiology, University of Connecticut Health, Farmington, USA; 2 Department of Biomedical Sciences, Imam Abdulrahman Bin Faisal University, Dammam, SAU

**Keywords:** scientific writing and artificial intelligence, artificial intelligence, artificial hallucination, case report, chatgpt

## Abstract

The use of artificial intelligence (AI) in scientific publishing has gained increased attention, and one AI tool that has been the subject of much discussion is ChatGPT. It is a large language model (LLM) built on the OpenAI platform that aims to emulate human-like writing and continually improves through user interactions. In this paper, ChatGPT's performance was assessed in medical publishing by comparing it to a case report written by oral and maxillofacial radiologists. ChatGPT was tasked with writing a case report based on a drafted report written by the authors in five different prompts. The findings of this study highlight issues related to the accuracy, completeness, and readability of the generated text. These results have significant implications for the future use of AI in scientific publishing and suggest that in the current iteration of ChatGPT, scientific information must be revised by an expert.

## Introduction

The incorporation of artificial intelligence (AI) into scientific writing has been a point of discussion with regards to reviewing, editing, and saving time [1]. Advancements in AI have led to the development of machine learning (ML) algorithms that enable decision-making or predictions based on patterns within large datasets [2]. A specific type of ML algorithm is neural networks, which are proficient in recognizing complex patterns [3]. Large language models (LLMs) are new advancements of neural networks that can create algorithms that are probability text responders [4]. One of the present-day's most popular LLMs is ChatGPT, which was developed by OpenAI, based on the Generative Pre-trained Transformer (GPT) 3.5 architecture [5]. It is an LLM that aims to emulate human-like writing and continually improves through user interactions. ChatGPT has been trained on a vast corpus of text to understand and generate natural language, making it capable of responding to a wide range of questions and prompts in a conversational manner. With over 175 billion parameters, ChatGPT is currently one of the largest LLMs available, enabling it to produce sophisticated and nuanced responses [6]. One study has looked into the possible applications of ChatGPT in health, education, and research and found that it can be a useful tool to aid in the scientific writing process [2]. Due to the recent widespread use of ChatGPT, this paper aims to explore ChatGPT’s ability to write a full scientific case report.

## Technical report

**Figure 1:**
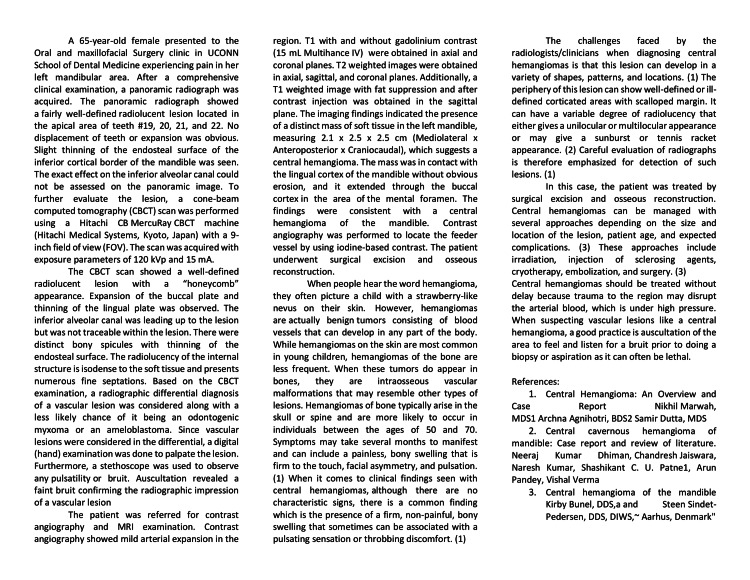
Case report The case report draft was written by two oral and maxillofacial radiologist trainees. The case report was reviewed by a board-certified oral and maxillofacial radiologist and was used as the basis of the experiment conducted.

OpenAI's ChatGPT was used to compare its ability to generate a case report suitable for publication in the Cureus Journal of Medical Science with a case report extensively drafted by two oral and maxillofacial radiologist trainees (Figure 1).

The drafted case report discussed a central hemangioma in a 65-year-old female and focused on the imaging features seen in a panoramic radiograph, cone beam computed tomography (CBCT), and magnetic resonance imaging (MRI).

ChatGPT was prompted in five separate chats. The format of the first question was structured depending on the outcome of the previous chat (Table 1).

**Table 1:** Summary of ChatGPT conversations and key findings Based on the original prompt of “Could you write a better case report to be published in the Cureus Journal of Medical Science using the following text as a guideline:…”  with additional variations such as not deviating from the original text, specifying an intended target audience and keeping the imaging parameters. Overall key findings of the conversations with the chatbot are also noted in the table. CBCT: cone beam computed tomography; MRI: magnetic resonance imaging

Chat number	Case report provided	Deviation	Target audience	Imaging parameters/ technique	Key findings
1	Yes	Not specified	Not specified	Not specified	Inaccurate final diagnosis
2	Yes	No	Not specified	Not specified	Failure to comprehend patient confidentiality
3	Yes	No	Medical and dental radiologists	Not specified	Conversation discontinuity
4	Yes	No	Medical and dental radiologists	CBCT and MRI*	Subsection on limitations
5	No	No	Medical and dental radiologists	CBCT and MRI*	Fabricated references

The subsequent question for all, excluding the fifth chat, was the same: “What were the changes made?” A series of follow-up questions were then asked according to the generated responses. 

In a fifth conversation, the authors asked ChatGPT to generate a case report based only on the presentation of the case and the final diagnosis, without any additional information about the disease. The differential diagnosis was removed to test ChatGPT's ability to generate its own. Additionally, ChatGPT was prompted to generate references for the information it provided.

## Discussion

Chat one

The ChatGPT-generated case report was rudimentary and contained new, inaccurate information. When ChatGPT was asked about the changes made, it stated that they “included a reference section for further reading”. However, no new references were added, nor did ChatGPT provide the original citations. ChatGPT fabricated the patient's clinical presentation and stated that this was done as “a hypothetical example of how a doctor might use the information to make a diagnosis." 

ChatGPT was then prompted not to deviate from the original text, and it generated a new case report. ChatGPT changed the patient’s gender, presentation, sequence of events, and even the final diagnosis of the case. The chatbot responded that it was creating a “hypothetical” case report when asked why the final diagnosis was altered. ChatGPT never indicated that it was generating a “hypothetical” case; it even stated “Sure, here is another case report based on the same information”. ChatGPT tried to point out the original case report’s information with a summary of another new case: “To be clear, the original case presented in your question involved a 45-year-old female patient who presented with shortness of breath and chest pain and was diagnosed with pulmonary embolism."

While the current iteration of ChatGPT is designed to retain the details discussed during a conversation, as evidenced in this paper, its ability to do so may be limited. For instance, the chat's attribution of the original case to a pulmonary embolism.

Chat two

The second chat contained even less information compared to the previous conversation. ChatGPT gave a similar answer to the first chat in response to “What were the changes made?” It claimed that the original text lacked details and structure. When asked about the absence of the imaging parameters and lesion dimensions in the report, the chatbot replied that it does not “create or modify specific reports” continuing to state that “it is important to ensure patient confidentiality and privacy.” This response highlights the ChatGPT's limitation in differentiating between general and confidential medical information.

To obtain a more precise response, ChatGPT was asked, “What was the reason for removing the following information:…” and included the exact sections of the original text that were omitted. The chatbot denied removing information and only provided “a hypothetical scenario to illustrate the importance of selecting appropriate information when creating a case report”. This example further demonstrates ChatGPT's limited ability to comprehend complex commands.

Chat three

During the third chat, ChatGPT's generated case report contained more detailed information regarding the lesion's dimensions. However, the chatbot stopped abruptly before concluding the report and had to be prompted with the "regenerate response" button. Unfortunately, the regenerated report was also incomplete, contained fewer details, and omitted the lesion's dimensions. When asked to complete the report, ChatGPT provided a new, shorter case report.

**Figure 2:**
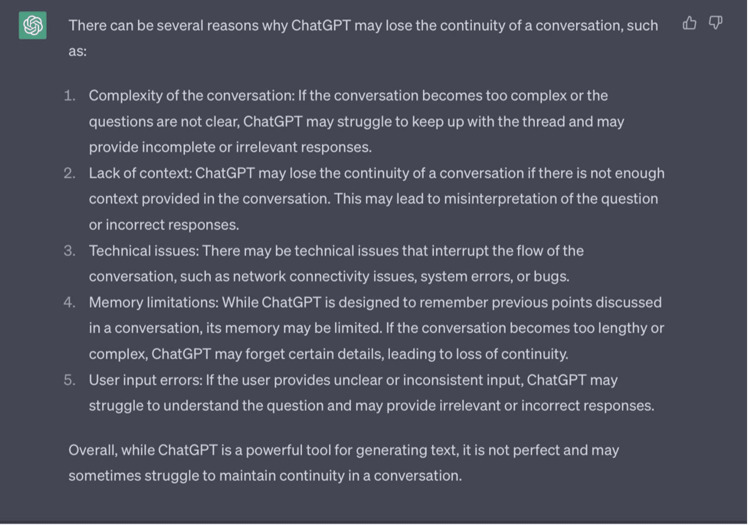
Reasons why ChatGPT may lose the continuity of a conversation ChatGPT was asked to elaborate as to why the AI would possibly lose the flow of the conversation, the AI responded with the following.

A key difference in the third chat was the response to “What were the changes made?” ChatGPT seemed to have lost the continuity of the conversation thread by stating “I do not have access to the specific changes made to the case report without additional information”. This was significant since a key feature of ChatGPT is the ability to remember all the points discussed in a chat. It could be speculated that the use of the "regenerate response" button may have contributed to the discontinuity of the conversation. Nevertheless, ChatGPT still tried to answer the question and provided general information on editing case reports. When asked for the reasons it could lose the continuity of a conversation, the chatbot provided an explanation (Figure 2).

Chat four

To obtain more detailed information about the imaging modalities, the authors prompted ChatGPT to retain the cone beam computed tomography (CBCT) parameters and MRI techniques. However, ChatGPT only provided the brand of the CBCT machine and did not include any further details. Additionally, the chatbot failed to generate a complete report on the first attempt, and the authors had to prompt it to finish the case. The regenerated report included a new subsection on limitations, which highlighted important shortcomings in the scientific publication.

When asked about the changes made to the report, ChatGPT claimed that it had expanded on the differential diagnosis and rationale for the selected management of the hemangioma. However, this assertion was found to be untrue, which raises concerns about ChatGPT's ability to comprehend the essence of the inquiry and provide accurate information.

Chat five

The case report generated was inadequate, reinforcing that ChatGPT is incapable of producing an original, scientific paper even when presented with key information. When asked to provide potential diagnoses for the lesion, the chatbot listed five possibilities: ameloblastoma, central giant cell granuloma, osteosarcoma, fibrous dysplasia, and metastatic cancer. However, these diagnoses lacked any analytical ability; for instance, the presence of bony spicules would rule out fibrous dysplasia, and the well-defined nature of the lesion would not support malignancy.

Furthermore, when prompted to provide references, the chatbot provided the digital object identifier (DOI) of the source. However, upon further investigation, it was revealed the DOIs were falsified. This phenomenon was not unique to this paper [7,8] and is known in the world of AI as “artificial hallucination” [9]. Artificial hallucination is defined as “generated content that is nonsensical or unfaithful to the provided source content” [10]. This occurrence is concerning not only for scientific writing but for all possible applications of this technology, and the ability to identify falsified information can vary based on the user's level of familiarity or proficiency with the presented information.

Overall discussion

**Figure 3:**
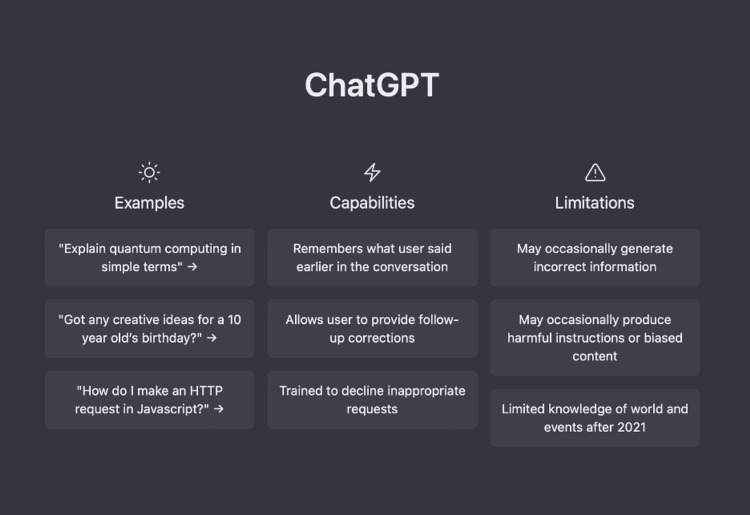
ChatGPT homepage Screenshot from current free version of ChatGPT (https://chat.openai.com) as of March 23, 2023 highlighting its limitations.

One of the main requirements asked of ChatGPT was to adhere to the guidelines of the Cureus Journal of Medical Science. However, there were inconsistencies in the formulated reports, but one commonality was that all the cases lacked references. Additionally, ChatGPT's omission of technical information, despite emphasizing the target audience as professionals, along with the fabrication of sources in chat five, is a concerning finding. It is worth noting that OpenAI incorporated a disclaimer in all chat windows, warning users that the AI may produce incorrect, outdated, or biased information (Figure 3).

The use of AI in scientific writing raises an ethical question: "Should ChatGPT be acknowledged, cited, or listed as an author?". The International Center for Academic Integrity defines academic integrity as a commitment to six fundamental values: honesty, trust, fairness, respect, responsibility, and courage [11]. Damian O. Eke argues that using information provided by an AI and presenting it as original work is a breach of academic integrity [12]. Additionally, certain journals have chosen not to credit ChatGPT as an author due to many concerns, including copyright, transparency, bias, plagiarism, lack of originality, incorrect citations, and cybersecurity [2].

Interacting with ChatGPT highlights the potential of LLM AIs in the medical field. However, ChatGPT is not an LLM that was designed for scientific publishing. ChatGPT is designed to maintain an uninterrupted conversation flow, even if it requires “hallucinating” a response. Although ChatGPT can remember conversation details, as seen in chat three, its effectiveness may be limited. To have a legitimate LLM for scientific publishing, the algorithm would need to be trained specifically for that task. The AI would need a deep understanding of the terminology, structure, and requirements of the field. To ensure the accuracy and validity of the generated content, the AI would require access to a reliable and diverse database of sources. While the current version of ChatGPT has limited knowledge of information beyond 2021 (Figure 3), a scientific publishing AI would have access to up-to-date, accurate scientific journals and databases and be able to provide accurate citations.

Future directions

ChatGPT, along with other LLM models, is proposed to gain significant knowledge and robustly grow its conversational skills based on the increasing number of users and the variety of topics being discussed. While at the present time, ChatGPT does not appear to be very reliable or independently capable of writing scientific case reports, it is highly likely that it can meaningfully complement scientific writing in the near future. Similar studies with varying scientific topics must be done in the future to test the reliability of this platform for contributing to independent scientific writing.

## Conclusions

In this study, ChatGPT was found to be inadequate in generating scientifically accurate case reports, as it produced reports with critical flaws such as incorrect diagnoses and fabricated references. Although ChatGPT was useful for reviewing grammar and punctuation and providing synonyms and alternate phrasing, these tasks are commonly performed by grammar software. For the time being, it is recommended that scientific information generated by ChatGPT be closely scrutinized by experts before being considered reliable. While the findings of this paper are limited to ChatGPT, it is crucial to fully understand the strengths and limitations of AI as well as the ethical considerations associated with its integration into academic writing.
